# Ambiguity Processing Bias Induced by Depressed Mood Is Associated with Diminished Pleasantness

**DOI:** 10.1038/s41598-019-55277-6

**Published:** 2019-12-10

**Authors:** Xiao-Xiao Lin, Ya-Bin Sun, Yu-Zheng Wang, Lu Fan, Xin Wang, Ning Wang, Fei Luo, Jin-Yan Wang

**Affiliations:** 1 CAS Key Laboratory of Mental Health, Institute of Psychology, Beijing, China; 2 Department of Psychology, University of Chinese Academy of Sciences, Beijing, China; 3 Sino-Danish Center for Education and Research, Beijing, China

**Keywords:** Emotion, Psychology, Depression

## Abstract

Depressed individuals are biased to perceive, interpret, and judge ambiguous cues in a negative/pessimistic manner. Depressed mood can induce and exacerbate these biases, but the underlying mechanisms are not fully understood. We theorize that depressed mood can bias ambiguity processing by altering one’s subjective emotional feelings (e.g. pleasantness/unpleasantness) of the cues. This is because when there is limited objective information, individuals often rely on subjective feelings as a source of information for cognitive processing. To test this theory, three groups (induced depression vs. spontaneous depression vs. neutral) were tested in the Judgement Bias Task (JBT), a behavioral assay of ambiguity processing bias. Subjective pleasantness/unpleasantness of cues was measured by facial electromyography (EMG) from the zygomaticus major (ZM, “smiling”) and from the corrugator supercilii (CS, “frowning”) muscles. As predicted, induced sad mood (vs. neutral mood) yielded a negative bias with a magnitude comparable to that in a spontaneous depressed mood. The facial EMG data indicates that the negative judgement bias induced by depressed mood was associated with a decrease in ZM reactivity (i.e., diminished perceived pleasantness of cues). Our results suggest that depressed mood may bias ambiguity processing by affecting the reward system.

## Introduction

We face ambiguous information on a daily basis. Imagine you are sitting in a bar and you notice an attractive man/woman smiling at you. Is he/she expecting you to come approach? Or is he/she laughing at your clothing style? A positively biased interpretation may make you feel confident and good, while a negative interpretation may lead to self-doubt and frustration. Thus, biases in ambiguity processing can have consequences for both mood and behavior.

Depressive disorders are featured by sad or depressed mood as well as maladaptive cognitive changes^1^. One of the most prominent cognitive changes in depression is the tendency to process emotionally ambiguous information in a negative/pessimistic manner. Negative biases in perception of neutral/ambiguous facial expressions^2–4^, attribution of others’ behaviors^5,6^, and more commonly, interpretation of self-relevant ambiguous stimuli^7,8^ have been consistently observed in spontaneous and/or induced depressed mood. Although debatable, there is also evidence that these biases may play a causal role in depression^9,10^. For example, interventions that successfully eliminate these biases have been found to effectively reduce depressive symptoms^10^ (but see^11^).

It has been implied that depressed mood can exacerbate ambiguity processing biases^12,13^, but the underlying mechanisms are not fully understood. Theoretically, depressed mood may affect the processing of ambiguous information both indirectly and directly. Several cognitive theories of depression have suggested that depressed mood can prime dysfunctional higher cognition and/or mood-congruent memories, making negative meanings of an ambiguous stimulus more accessible, and thus indirectly bias the disambiguation^14,15^. Prior studies have therefore employed sad mood induction to facilitate the detection of these biases^15,16^. Alternatively, individuals may rely on subjective emotional feelings (e.g., perceived pleasantness/unpleasantness) about ambiguous information which may be directly influenced by depressed mood. Comparative behavioral studies have suggested that low mood states (e.g., depression, sadness) induced by risky and unpredictable environments might “down-regulate” the pleasantness and expectation of rewards, which might be associated with negatively biased processing of ambiguous cues^17,18^. Similarly, evidence from social psychology implies that mood states may alter perceived pleasantness/unpleasantness of materials/events, making incidental moods “an implicit perceptual lens for interpreting” unrelated events^19,20^. Hence, when there is limited objective information for the disambiguation of a presented cue, perceived emotional feelings arising from the current mood state can serve as a source of information. In contrast, the alteration in perceived feelings does not necessarily affect the judgements of unambiguous or less ambiguous cues where objective information is available. As loss of interest and pleasure in usual activities (i.e., anhedonia) is a core feature of depression^21,22^, ambiguous cues may also be perceived as less pleasant and as such will be judged negatively.

If a depressed mood, as argued above, has a direct impact on ambiguity processing biases by altering perceived emotional feelings, then clinical interventions should pay more attention to the reciprocal relationship between mood and these cognitive biases. For example, it would be beneficial to targeting depressed mood at the same time as modifying negative biases (e.g.,^23^), as depressed mood might make these biases more resistant to “pure” cognitive interventions. Indeed, studies have suggested that when in a negative mood state, people may have more difficulties disengaging from negative self-referent information, thus hindering cognitive reappraisal^24,25^.

Despite the potential impact of perceived emotional feelings on ambiguity processing, empirical studies examining this hypothesis are limited. One reason might be the lack of appropriate paradigms. Traditional paradigms utilize verbal or social cues to probe ambiguity processing biases^7^. Unfortunately, this approach is not adequate for the present purpose, as verbal and social cues are inherently emotional. For example, the ambiguous word “ceremony” can be perceived as more pleasant by those who recently participated in a wedding than those who participated in a funeral. Such individual differences in the “inherent” emotional tone of cues would confound those relevant to the current mood. An ideal paradigm for the present purpose should utilize neutral cues that only gain emotional valence in the context of the task itself. More recently, researchers have developed the Judgement Bias Task (JBT) as a cross-species behavioral assay of ambiguity processing bias^26–28^, which involves judging presented nonverbal ambiguous cues as predicting reward or punishment. Subjects first learn the associations between reward/punishment and previously neutral cues, i.e., pure tones. Once the association was learnt, they are then presented with intermediate frequency tones as ambiguous cues. Participants have to infer the emotional meanings of ambiguous cues based on specific rules (e.g., tones acoustically more similar to rewarding tone signals reward). Thus, a positive/negative judgement bias is measured by preferentially judging ambiguous cues as predicting reward or punishment respectively. Studies have reported associations between negative judgement bias and low mood^29^ and depressive rumination^30,31^.

Our working hypothesis was that depressed mood would affect ambiguity processing by altering perceived emotional feelings of cues. If so, then an induced depressed mood should result in a negative bias in judgement of ambiguous cues, and more importantly, perceived pleasantness/unpleasantness of cues should mediate the relationship between mood states and judgement bias. To test whether depressed mood has a direct impact on ambiguity processing, we experimentally manipulated mood states in two groups of participants (sad vs. neutral). A group of spontaneous dysphoric individuals (defined by elevated depressive symptoms) was also included as a positive control group to verify whether the ambiguity processing bias resulted from depressed mood induction was similar to that in naturally occurring depression. The three groups (induced depression vs. spontaneous depression vs. neutral) were tested and compared in the JBT.

As we predicted that individuals would use their current subjective emotional feelings (pleasantness/unpleasantness) as a source of information to judge ambiguous cues, and that a depressed mood would directly bias ambiguity processing by altering emotional feelings, perceived pleasantness or unpleasantness of each cue was measured by simultaneous facial electromyography (EMG) recordings from the zygomaticus major (ZM, “smiling”) and corrugator supercilii (CS, “frowning”) muscles. ZM/CS activity has previously been validated as a reliable physiological correlate of perceived pleasantness/unpleasantness in healthy participants^32,33^. Specifically, recent studies have shown that dysphoric and clinically depressed individuals exhibit blunted ZM activation in response to rewards which is associated with a decreased self-report reward liking, suggesting that ZM activity is a reliable marker of diminished perceived pleasantness (i.e. hedonic reactivity) in depression^34–36^.

## Methods

### Participants

One hundred and thirty-six healthy college students (86 females, age range 18 to 29 years) were recruited via advertisement. We initially recruited 83 participants based on an *a priori* power analysis (supplementary material S1), and 53 additional participants were recruited based on reviewer comments. The advertisement described the purpose of the experiment as a study of auditory perception and indicated that those majoring in music or having formal musical training were not accepted. All participants were Chinese, right-handed, had normal or corrected to normal vision, normal hearing, and reported no history of neurological/psychiatric disorders. No participant majors in music, neither did they have formal musical training. Twenty-nine participants (20 females) with Beck Depression Inventory-II (BDI-II) scores >13 were assigned to the dysphoric group, and the remainder of the participants were randomly assigned to either the sad-mood induction group (n = 53, 35 females) or the neutral-mood group (n = 54, 32 females). All procedures performed in this study were approved by the Research Ethics Committee of the Institute of Psychology, Chinese Academy of Sciences and were in accordance with the Declaration of Helsinki of 1975, as revised in 1983. All participants gave written informed consent before participation.

### Materials and Procedures

#### Measures

Depressive symptoms were assessed by the BDI-II which has been validated and widely used in the Chinese population in previous studies e.g.,^37,38^. A cut-off 13 was used as a criterion for mild depression. The Cronbach’s alpha of the BDI-II in the current study was 0.89.

Depression-linked dysfunctional cognition was measured by the **Dysfunctional Attitudes Scale (DAS)**, which has been validated and widely used in the Chinese population in previous studies^39^. To examine whether dysfunctional attitudes were affected by the experimental procedure, two 9-item short forms of the DAS (SF1 and SF2)^40^ were administrated before and after the experiment in a counterbalanced order. The two short forms have demonstrated good internal consistency (0.84 and 0.83, respectively)^40^. In the present study, however, the Cronbach’s alpha of the DAS-SF1 and DAS-SF2 was 0.68 and 0.77, respectively.

Ruminative coping and anxiety have been found to be associated with bias score in the JBT^31^. To control for ruminative coping and anxiety, a 10-item subset of the **Ruminative Responses Scale (RRS)**^41^ and the **State-Trait Anxiety Inventory (STAI)**^42^ were administrated. The 10 items from the original RRS have been widely used to measure the two subcomponents of rumination, namely brooding and reflection (or reflective pondering) e.g.,^43^. A previous study^44^ suggested that brooding may be specifically related to depression and constitutes the maladaptive component of rumination. In contrast, reflection may be an adaptive component of rumination. In the present study, the brooding subscale internal consistency was 0.73 and the reflection subscale internal consistency was 0.74. The overall internal consistency of the RRS subset was 0.81. The STAI is a 40-item inventory that measures both state and trait anxiety (20 items for each). In the present study, the Cronbach’s alpha for the state anxiety and the trait anxiety was 0.91 and 0.92, respectively.

An 11-point visual analogue mood scale (VAS) was administrated four times (pre-test, before mood induction, after mood induction, and post-test), with + 5 = “very happy”, 0 = “neutral”, and −5 = “very sad”. Participants were instructed to “select a point on the scale that reflects your current mood”.

#### Mood manipulation

An approximately four-minute video clip from the Chinese film *Aftershock* (唐山大地震, 2010, IMDb: tt1393746) was chosen for the induction of sad mood. The clip depicts a mother, who is faced with the choice of giving up either her 8-year old daughter or her 7-year old son to save the other after losing her husband in an earthquake. She chooses to save the son and leave the daughter to die. She cries loudly after seeing her daughter’s dead body. This clip was chosen because it portrays great personal loss; one of the most prominent causes of sad mood in daily life. Participants were instructed to “get involved in the mood shown by the main character as deeply as you can”, and “if any mood is evoked, do not suppress it, and feel free to express it”. A different video clip (also approximately 4 minutes long) from a popular Chinese documentary *I Repair Cultural Relics in the Palace Museum* (我在故宫修文物, 2016, IMDb: tt6793448) was shown to participants in the neutral group with the same instructions. This video clip depicts the tranquil routine work of experts who repair cultural relics.

As the dysphoric individuals were already in a depressed mood, we did not administrate mood induction procedure to them. To control for the potential impact of video watching on the subsequent task, they watched the neutral clip but were simply instructed to “watch with concentration”.

#### The judgement bias task

**Figure 1:**
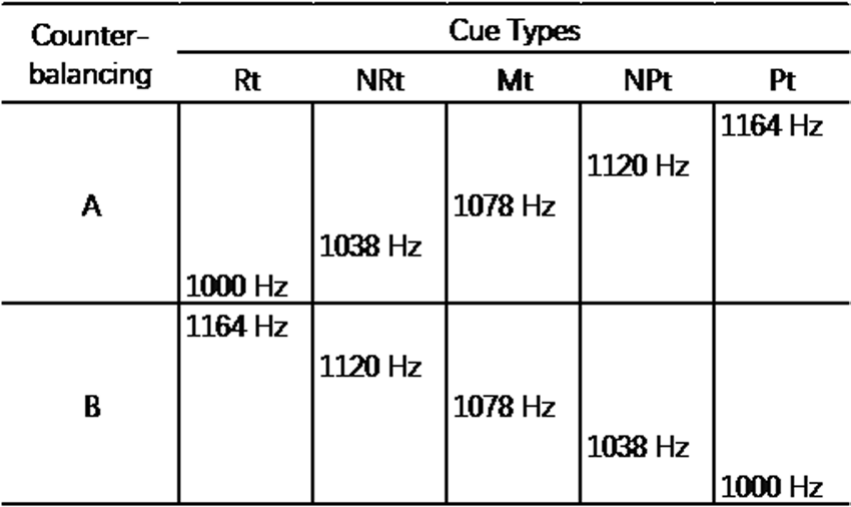
Reference cues and ambiguous cues. Five sinusoidal tones with gradually rising pitch were presented (frequency: 1000 Hz, 1038 Hz, 1078 Hz, 1120 Hz, and 1164 Hz; separated by 0.25 bark). The pitches were selected according to the Bark Frequency Scale^60^ so that any pair of two adjacent tones are perceived as acoustically equidistant. The same parameters have been used in previous JBT studies^30,31^. A pilot study confirmed the effectiveness of the acoustic parameters (data not presented). Participants were counterbalanced into two groups. For half of participants (row A), the highest pitch was the rewarding tone (associated with monetary reward, Rt) and the lowest was the punishing tone (associated with monetary punishment, Pt), and for the other half (row B) this was reversed. Rt: rewarding tone; NRt: near-rewarding tone; Mt: middle tone; NPt: near-punishing tone; Pt: punishing tone.

Auditory cues: The JBT was adopted from previous studies^30,45^. As shown in Fig. 1, five tonal cues of gradually rising pitch were presented. The highest and lowest pitches serve as reference tones, one associated with monetary reward (rewarding tone, Rt) and the other associated with monetary punishment (punishing tone, Pt). The three intermediately pitched tones are ambiguous cues, labeled as near-rewarding tone (NRt), middle tone (Mt), and near-punishing tone (NPt).

**Figure 2:**
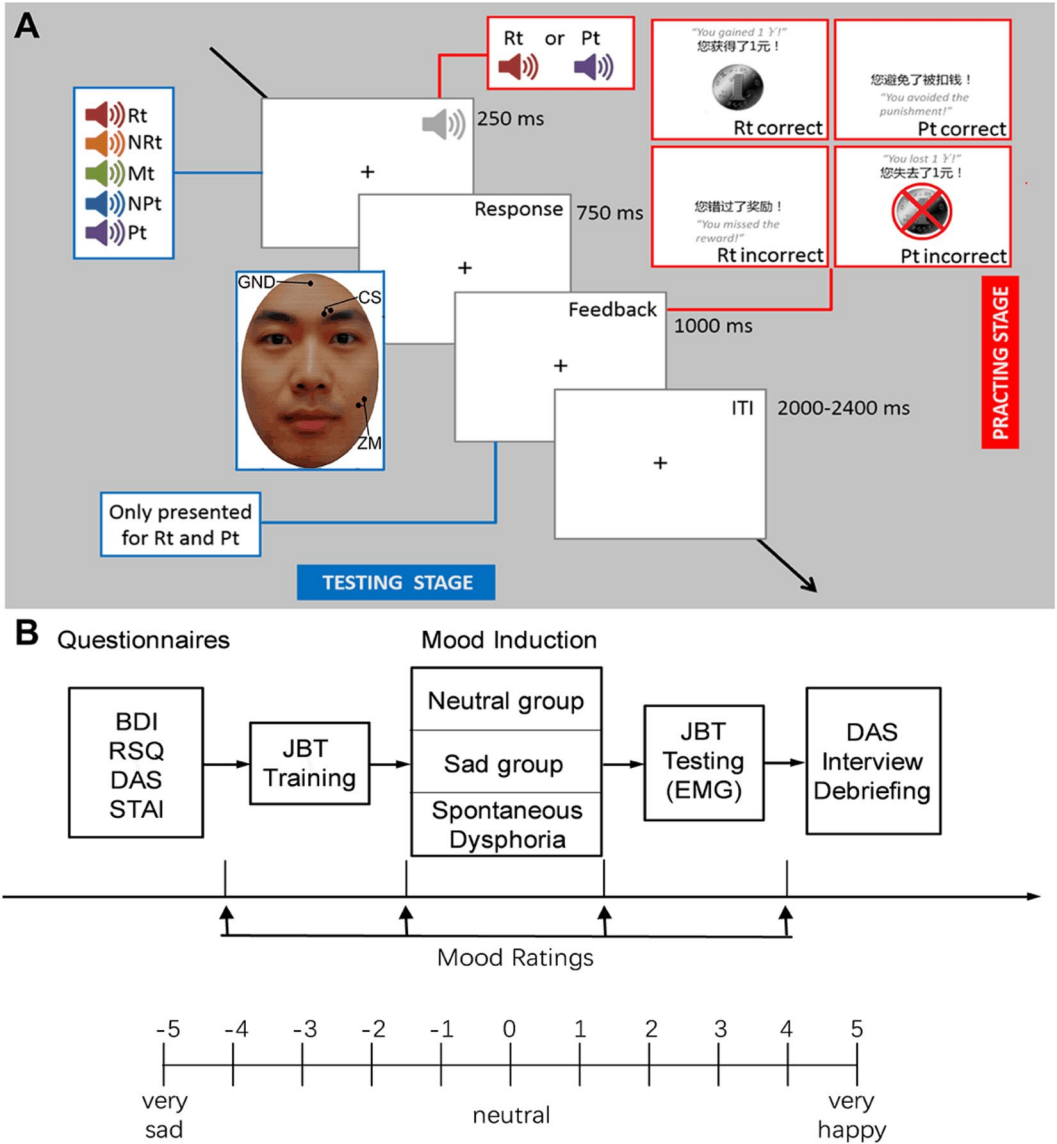
*Experimental procedure*. (**A**) Schematic representation of the Judgement Bias Test (JBT). The JBT consists of a training stage (red) and a test stage (blue). Each trial was comprised of a tone (auditory cue) of 250 ms followed by a response window of 750 ms. Feedback was presented for 1,000 ms duration following the response window. Perceived pleasantness and unpleasantness in response to cues were recorded simultaneously from the ZM (“smiling”) and CS (“frowning”) muscles using facial EMG. The face in this illustration was generated using software (FantaMorph5; www.fantamorph.com). Feedback was only presented for Rt and Pt and consisted of Rt correct, Rt incorrect, Pt correct or Pt incorrect. The inter-trial interval (ITI) varied randomly between 2,000 to 2,400 ms (2,200 ms on average). Screenshots of the four types of feedback are presented in the upper right corner. Rt: rewarding tone; NRt: near-rewarding tone; Mt: middle tone; NPt: near-punishing tone; Pt: punishing tone. (**B**) Schematic representation of the general experimental procedure.

Task procedure: As shown in Fig. 2A, the test procedure consisted of a training stage and a testing stage. In each trial, participants had to identify whether a cue predicted reward or punishment by pressing one of two buttons (the REWARD or the PUNISHMENT button) on a keyboard with their left or right index finger (counterbalanced across participants).

Participants first learned the emotional meanings of Rt and Pt in the training stage (see supplementary material S1 for additional details). Correctly identifying Rt (pressing REWARD) resulted in a monetary reward (+1¥), while misidentifying (pressing PUNISHMENT) or omission (no pressing) resulted in no reward; on the other hand, correctly identifying Pt (pressing PUNISHMENT) avoided monetary punishment while misidentifying (pressing REWARD) or omission resulted in a punishment (−1¥). To reinforce the associations between reference cues and emotional meanings, responses were followed by feedback to inform participants of the outcomes (reward gained, reward missed, punishment avoided, or punishment incurred). Participants who failed to reach 80% accuracy after three repeated rounds of training were excluded from the study. This exclusion criterion was determined on the basis of previous studies e.g.^45^ as well as our piloting.

In the subsequent testing stage, NRt, Mt, and NPt were presented along with Rt and Pt. Participants were instructed to infer the emotional meanings of these new cues by the following rules: a new cue acoustically more similar to Rt/Pt also predicted reward/punishment. As responses to NRt, Mt, or NPt were not reinforced by feedback, these cues are ambiguous. The NRt and the NPt are partially ambiguous, as they can be disambiguated by objective physical features (acoustically more similar to either Rt or Pt). The Mt is fully ambiguous; it is acoustically equidistant between the Rt and the Pt, and therefore no physical feature can be used to disambiguate it.

The testing stage consisted of three blocks, each containing 60 trials (12 trials for each tone), resulting in a total of 180 trials. Trial order was randomized for each individual.

Index of ambiguity processing bias: Pressing the REWARD/PUNISHMENT button in response to a cue indicated judging the cue as predicting reward/punishment. A judgement bias score was calculated for each cue by subtracting the proportion of *judging it as predicting punishment* from that of *judging it as predicting reward*.

In the current study, ambiguity processing bias was operationally defined as the judgement bias score of the Mt (the fully ambiguous tone; indexing the magnitude of judgement bias), and that of the NRt and the NPt (the less ambiguous tones; indicating whether judgement bias generalized to less ambiguous events). A positive/negative bias score of the Mt indicates the tendency of preferentially judging a fully ambiguous cue as predicting reward/punishment.

### Facial electromyography recording and data reduction

During the JBT, electromyography (EMG) signals were recorded simultaneously from the left CS and ZM muscles following a standard procedure^46^ with Biopac MP150 system and Acqknowledge 4.3 (Biopac System Inc.). Participants were instructed not to talk or move their heads during recording. To conceal the purpose of the experiment, participants were told that the recording was to monitor the electrical activity of the brain (rather than facial muscles).

Parameters of data collection and raw data preprocessing were adopted from Sun, *et al*.^47^. Data were processed using MATLAB (www.mathworks.com). Continuous data were segmented from 0.4 seconds before and 1.0 second after auditory cue onset. For each participant, trials with a baseline amplitude greater than 2 SDs from the mean amplitude of all baselines were eliminated^48^. To acquire reliable averaged EMG signals, we require a minimum of 25 trials for each type of cue. Therefore, participants with more than 30% eliminated trials from any one type of cue were excluded from subsequent EMG analysis. Mean EMG amplitudes during each 100-ms time bin were expressed as a percentage change from the baseline to standardize individual differences in absolute EMG amplitudes.

### General procedure

A schematic diagram of the general procedures is shown in Fig. 2B. Upon arrival, participants were led into an individual, soundproof room where they were seated and rested for 5 minutes. Participants were given consent forms to read and sign. Next, participants were given instructions on how to complete questionnaires (BDI-II, RRS, DAS-SF and STAI, presented on a laptop in the form of interactive webpages). After completing the questionnaires, participants entered the training stage of the JBT test, during which an experimenter scored their BDI-II. Non-depressed participants (BDI <14) were randomly assigned to either the neutral or sad group, while those with depressed mood (BDI ≥ 4) were assigned to the dysphoric group. Upon completion of the training stage, mood manipulation was administrated. All participants then proceeded into the testing stage of the JBT. After JBT testing, participants completed the second DAS-SF, were interviewed (supplementary material S2), thanked, paid, and dismissed.

### Data analysis

Two participants from the dysphoric group and one from the neutral group were excluded from behavioral data analyses as they failed to reach accuracy criteria for the JBT. Twelve participants (four from the neutral group, six from the sad group and two from the dysphoric group) were excluded from EMG analysis; ten were excluded according to the exclusion criteria described above, and two were excluded due to equipment malfunction. The final sample size was N = 133 for behavioral data analysis, and N = 121 for EMG analysis.

Data were analyzed using STATISTICA 8.0 software (StatSoft Inc., Tulsa, OK, USA). Prism 5.0 (GraphPad Sofware Inc., La Jolla, CA, USA) was used to generate graphs. Group differences in demographic characteristics were assessed by one-way ANOVA and chi-square test. Effectiveness of mood manipulation was examined by a two-way (group × time) repeated-measure ANOVA on the VAS.

Central to our hypothesis were the mood effects on judgement bias, and the following confirmatory analyses were planned. A two-way (group × tone) repeated measures ANOVA were conducted on bias scores. To examine mood effects on perceived emotional feelings of cues, three-way (group × tone × time) repeated measures ANOVAs were conducted for cue-elicited CS and ZM muscle activity, separately. If a significant three-way interaction was observed, we would examine the group × time interaction within the tone condition by conducting a two-way ANOVA. As we predicted emotional feelings would mediate the effects of depressed mood on judgement bias, a mediation analysis was performed using PROCESS macro for SPSS^49^, with mood state (group) as the independent variable, perceived pleasantness as the mediator (mean percentage change of ZM EMG during the 1,000 ms epoch after cue onset), and judgement bias (bias score of Mt) as the dependent variable. A bias-corrected bootstrap analysis was conducted with 5,000 resamples. Unstandardized coefficients were reported as recommended by Hayes^50^. To correct for multiple comparisons, false discovery rate (FDR) adjusted p-values were computed for these analyses following the Benjamini–Hochberg procedure using R (http://www.R-project.org/).

To further validate the direct effect of depressed mood induction on the judgement of ambiguous cues, supplementary ANCOVAs were performed to control for the potentially confounding variables. These ANCOVAs are exploratory, and the results are reported in the Supplementary Materials. As argued above, depressed mood might also indirectly affect ambiguity processing by activating depression-linked dysfunctional cognitions. Moreover, previous studies have indicated a correlation between negative judgement bias and depressive rumination and anxiety^31^. Hence, ANCOVAs using demographic characteristics, RRS, and STAI as covariates were conducted whenever applicable. The DAS, a measure of depressive dysfunctional cognitions, was assessed both before and after experiment because several studies suggest that depressed mood induction may aggravate state-like dysfunctional attitudes in those with greater sensitivity to emotional stress (e.g.^51^). We had planned to include the DAS in supplementary ANCOVAs, but the internal consistency of the DAS-SF1 for the present study was too low (<0.70). Nevertheless, the inclusion of the DAS made no substantial change to the results of these ANCOVAs.

Data normality was examined by the Shapiro-Wilk test. For ANOVAs, Greenhouse-Geisser correction was applied if data failed the Mauchly’s test of sphericity. Bonferroni corrections were applied to post-tests, and p-values reported in these post-tests were adjusted as follows: corrected p-value = observed (uncorrected) p-value × number of comparisons made. The alpha level for all statistical tests was set at 0.05.

## Results

### Participant characteristics

The three groups (sad, neutral and dysphoric) did not differ in age (one-way ANOVA, *F*_2,130_ = 0.92, p = 0.40) or gender (chi-square test, χ^2^ = 1.974, *p* = 0.37). The neutral group and the sad group did not differ in any pre-test self-report measures (Bonferroni post-test, all *p*s > 0.38). The dysphoric group reported more depressive symptoms, more dysfunctional cognitions, greater (state and trait) anxiety, more depression-related ruminative thoughts, and sadder mood VAS ratings at baseline, as compared with both the sad and neutral groups (Bonferroni post-test, all *p*s < 0.05) (Table 1), suggesting that they were in a depressed-mood state. At the end of the experiment, the dysphoric group also gained significantly less money compared with both the sad group and neutral group (all *p*s < 0.05), while the sad group did not differ from the neutral group (Table 1).

**Table 1:** Participant characteristics.

	Neutral (n = 53)		Sad (n = 53)		Dysphoric (n = 27)	
	M	SD	M	SD	M	SD
Mean age, year	22.6	2.4	22.1	2.3	21.9	2.4
Female, n	31	—	35	—	20	—
BDI-II	5.2	3.7	4.7	4.3	21.8^a,b^	8.5
DAS-pre	18.8	3.7	17.7	3.4	22.1^a,b^	3.8
DAS-post	21.9	4.6	20.7	3.5	24.5^a,b^	3.4
STAI						
State anxiety	32.6	5.9	33.6	7.7	46.2^a,b^	12.4
Trait anxiety	37.7	7.4	39.3	9.0	51.4^a,b^	11.0
Rumination	21.5	4.5	21.7	4.4	25.1^a,b^	4.7
Brooding	10.5	2.4	10.7	2.7	12.9^a,b^	2.7
Reflection	11.0	2.7	11.1	2.6	12.2	2.9
Mood (baseline)	1.4	1.6	1.5	1.7	−1.3^a,b^	2.0
Net earnings*, Ɏ	55.9	13.2	54.3	12.0	44.2^a,b^	21.8

One-way ANOVA with Bonferroni post-test was conducted to compare means between three groups (Welch test was applied if the assumption of homogeneity of variances was not satisfied). For gender, chi-square test was performed.

BDI-II = Beck Depression Inventory-II; DAS = Dysfunctional Attitudes Scale (Short Form); STAI = State-Trait Anxiety Inventory.

a: *p* < 0.05, contrasted with neutral group; b: *p* < 0.05 contrasted with sad group.

*The average earnings from the JBT is around ¥50 (equals to about 8 U.S. Dollars).

### Mood manipulation

**Figure 3:**
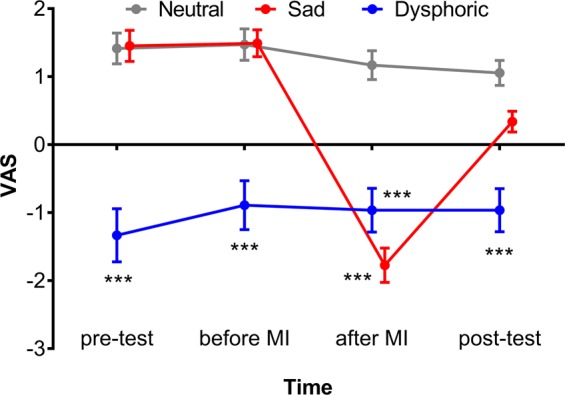
*Results of mood ratings*. Mood states were assessed four times throughout the experiment by the visual analogue mood scale (VAS). A two-way repeated-measure ANOVA revealed significant main effects of time and group, as well as a significant group × time interaction. Bonferroni post-tests indicated that participants who were exposed to the sad movie clip rated mood more negatively than those exposed to the neutral movie clip, indicating that the mood manipulation was successful. Dysphoric participants, on the other hand remained depressed (as demonstrated by a consistently low VAS score) throughout the experiment. Neutral group, n = 53; Sad group, n = 53; Dysphoric group, n = 27. MI = mood induction. ***p < 0.001, all vs. Neutral.

A two-way repeated-measures ANOVA was conducted on self-reported mood ratings to examine whether mood manipulation was successful (Fig. 3). There were significant main effects of group (*F*_2,130_ = 27.10, *p* < 0.001, ${\eta }_{p}^{2}=0.29$, observed power >0.99) and time (*F*_2.04,264.49_ = 32.30, *p* < 0.001, ${\eta }_{p}^{2}=0.20$, observed power >0.99) and a significant group × time interaction (*F*_4.07,264.49_ = 33.01, *p* < 0.001, ${\eta }_{p}^{2}=0.33$, observed power >0.99). A Bonferroni post-test found no significant differences between the sad group and the neutral group prior to mood induction (all *p*s >0.90). After mood induction, participants exposed to the sad movie clip rated mood more negatively on the VAS than those exposed to the neutral movie clip (mean −1.77 vs. 1.17, *p* < 0.001), indicating that mood manipulation was effective. The dysphoric group maintained a depressed-mood state throughout the experiment, as evidenced by persistent lower ratings on the VAS than the neutral group (Bonferroni post-test, all *p*s < 0.001), although both groups watched the neutral movie.

### Effects of mood states on the judgement bias Task (JBT)

**Figure 4:**
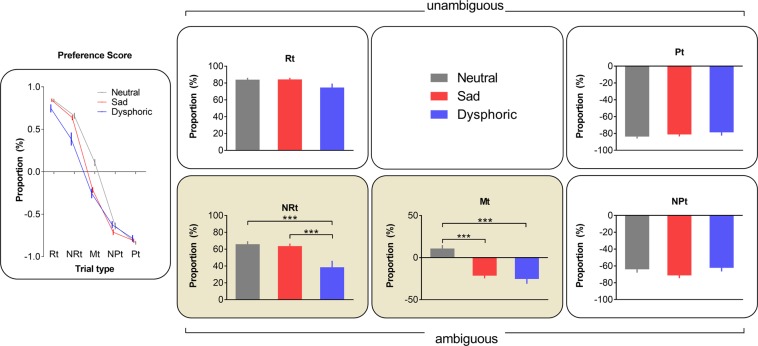
*Group difference in response to cues of different degrees of ambiguity*. Bias scores were calculated for each tone by subtracting the proportion of negative responses from the proportion of positive response. Group differences in bias scores were found on the ambiguous Mt and NRt, but not on the unambiguous Rt or Pt. Rt: rewarding tone; NRt: near-rewarding tone; Mt: middle tone; NPt: near-punishing tone; Pt: punishing tone. Neutral group, n = 53; Sad group, n = 53; Dysphoric group, n = 27. ***p < 0.001.

To examine mood effects on ambiguity processing, a two-way repeated-measures ANOVA of group × tone on bias score was conducted (Fig. 4A). A significant group × tone interaction and main effects of tone and group were found (group × tone interaction, *F*_5.35,347.76_ = 10.04, *p* < 0.001, FDR-corrected *p* < 0.001, ${\eta }_{p}^{2}=0.13$, observed power >0.99; tone effect, *F*_2.67, 347.76_= 1311.65, *p* < 0.001, FDR-corrected *p* < 0.001, ${\eta }_{p}^{2}=0.91$, observed power >0.99; group effect, *F*_2,130_ = 9.70, *p* < 0.001, FDR-corrected *p* < 0.001, ${\eta }_{p}^{2}=0.13$, observed power >0.95). Bonferroni test revealed that the three groups did not differ in the judgement of unambiguous cues (Rt and Pt). Importantly, both the dysphoric group (mean bias score: −0.25) and the sad group (mean bias score: −0.21) preferentially judged the fully ambiguous cue (Mt) as predicting punishment in contrast to the neutral group (mean bias score: 0.11, all *p*s < 0.001) (Fig. 4B), confirming the presence of a negative judgement bias under the induced sad mood and the spontaneous dysphoric mood. Furthermore, the dysphoric participants were more likely to judge the less ambiguous near-rewarding cue (NRt) as predicting punishment compared to both the neutral and the sad group (all *p*s < 0.001) (Fig. 4B). These results suggest that, compared with the sad group, the dysphoric group showed a generalized bias to negatively judge the less ambiguous near-rewarding cues as well as the fully ambiguous cue.

No significant group difference on other tones were found. There was no substantial change after controlling for covariates, except that the group main effect became insignificant (supplementary material S3).

### Effects of mood states on perceived pleasantness/unpleasantness of cues

To determine whether mood states altered perceived pleasantness/unpleasantness of cues, three-way group × tone × time repeated-measures ANOVAs were conducted on facial muscle activity for the ZM muscle and the CS muscle, separately.

**Figure 5:**
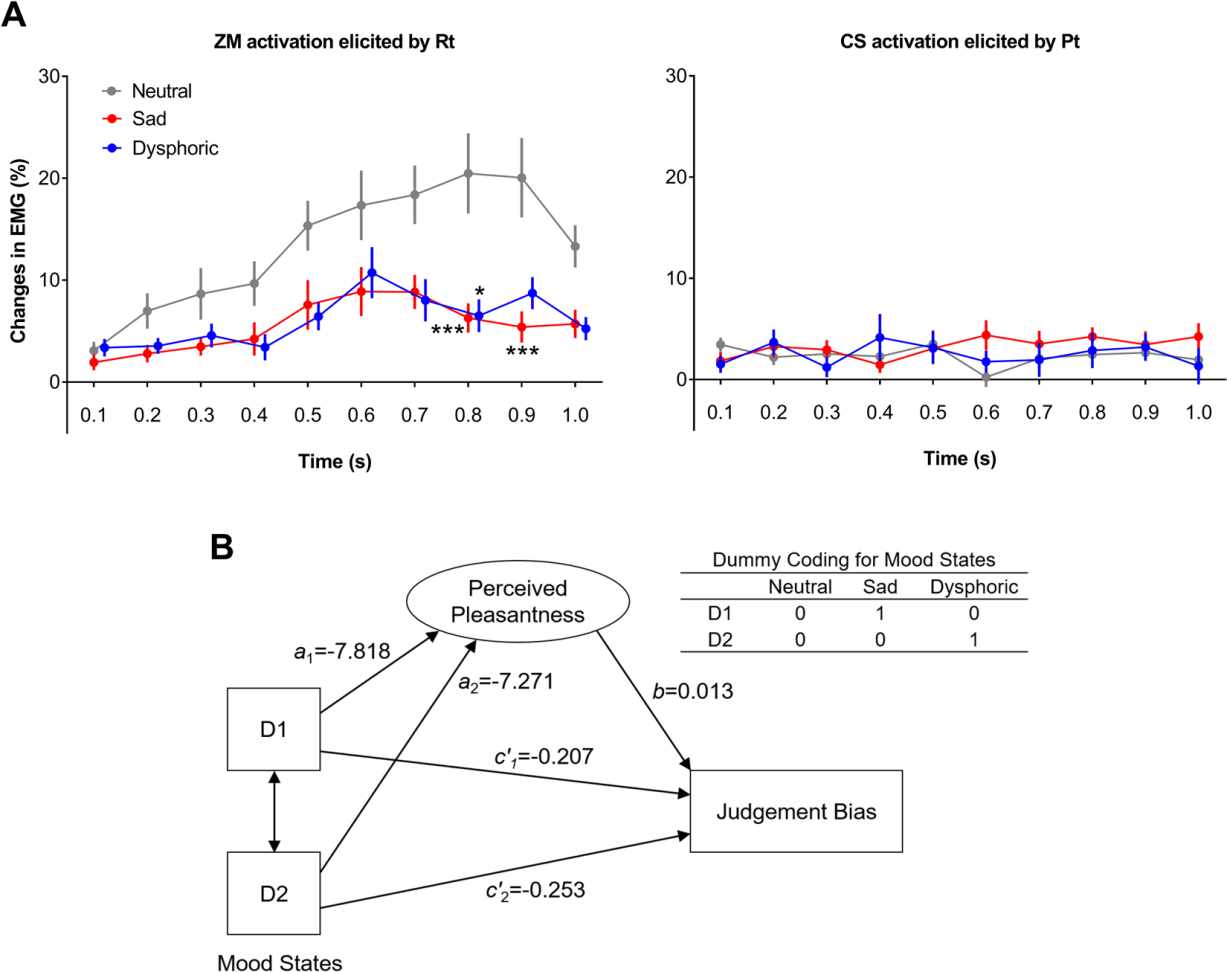
*Facial EMG recorded simultaneously during the JBT*. (**A**) Group difference in Zygomaticus Major (ZM) and Corrugator Supercilii (CS) activity in response to the rewarding tone (Rt) and the punishing tone (Pt). Neutral group, n = 49; Sad group, n = 47; Dysphoric group, n = 25. *p < 0.05, ***p < 0.001, all vs. Neutral. (**B**) Perceived pleasantness of cues mediated the mood effects on judgement bias. The multicategorical independent variable, mood state (group: neutral, sad, and dysphoric), was coded as dummy variables D1 and D2. The mediator (perceived pleasantness) was measured by mean ZM activity in response to the Rt. Both the sad mood group and the dysphoric group showed reduced pleasantness, which resulted in the negative judgement bias of ambiguous cues. Both experimentally induced sad mood (*a*_1_*b* = −0.10, 95% CI [−0.16, −0.05]) and naturally occurring dysphoria (*a*_2_*b* = −0.09, 95% CI [−0.15, −0.04]) had significant indirect effects on judgement bias through perceived pleasantness. All depicted paths are significant (all FDR-corrected *p*s < 0.05).

Significant changes in EMG were found for the ZM muscle (three-way interaction, *F*_19.77,1166.18_ = 2.15, *p* < 0.01, FDR-corrected *p* = 0.004, ${\eta }_{p}^{2}=0.04$, observed power > 0.99; tone × group interaction, *F*_4.50,265.40_ = 5.61, *p* < 0.001, FDR-corrected *p* < 0.001, ${\eta }_{p}^{2}=0.09$, observed power >0.95; tone × time interaction, *F*_9.88, 1166.18_ = 6.01, *p* < 0.001, FDR-corrected *p* < 0.001, ${\eta }_{p}^{2}=0.05$, observed power > 0.99; tone effect, *F*_2.25,265.40_ = 28.25, *p* < 0.001, FDR-corrected *p* < 0.001, ${\eta }_{p}^{2}=0.19$, observed power > 0.99; time effect, *F*_5.36,632.57_ = 12.53, *p* < 0.001, FDR-corrected *p* < 0.001, ${\eta }_{p}^{2}=0.10$, observed power >0.99) but not for the CS muscle. Closer inspection revealed that the Rt elicited the greatest ZM activity (i.e., pleasantness) than all other tones (Bonferroni post-test for tone effect, all *p*s < 0.001), while there was no significant difference in ZM activity elicited by other tones. The significant tone × group interaction suggested the three groups might differ in the magnitude of Rt-elicited ZM activation. Indeed, Rt-elicited ZM activation was significantly greater in the neutral group than in the sad group or the dysphoric group (Fig. 5A, left; two-way repeated measures ANOVA, group × time interaction: *F*_7.49,241.91_ = 2.67, *p* < 0.01, FDR-corrected *p* = 0.013, ${\eta }_{p}^{2}=0.04$, observed power > 0.99; time main effect: *F*_3.74, 241.91_= 13.00, *p* < 0.001, FDR-corrected *p* < 0.001, ${\eta }_{p}^{2}=0.10$, observed power > 0.90; group main effect: *F*_2,118_ = 7.09, *p* < 0.001, FDR-corrected *p* = 0.002, ${\eta }_{p}^{2}=0.11$, observed power > 0.90), suggesting that the sad mood and the dysphoric mood diminished the perceived pleasantness of cues. There was no substantial change in this pattern after controlling for covariates (supplementary material S3).

### Mediation effect of perceived pleasantness between depressed mood and judgement bias

To test whether the diminished pleasantness contributed to negative judgement bias, a mediation model was introduced. As shown in Fig. 5B, compared with neutral mood, experimentally induced sad mood (*a*_1_) and naturally occurring depressed mood (*a*_2_) decreased perceived pleasantness of cues, and participants with decreased pleasantness were biased to judge the ambiguous cue as punishment (*b*) (R^2^ = 0.43, *F*_3,117_ = 29.03, *p* < 0.001). Both experimentally induced sad mood (*a*_1_*b* = −0.10, 95% CI [−.16, −0.05]) and naturally occurring dysphoria (*a*_2_*b* = −0.09, 95% CI [−0.15, −0.04]) had significant indirect effects on judgement bias through perceived pleasantness. The model remained statistically significant after controlling for covariates (R^2^ = 0.46, *F*_10,110_ = 9.21, *p* < 0.001).

## Discussion

Depression is characterized by the presence of negative moods (sadness, emptiness, etc.) and diminished pleasantness in daily life (anhedonia). At the cognitive level, negative/pessimistic biases in ambiguity processing are well-established among people with depression. Evidence has suggested that negative moods in depression can exacerbate these biases. However, the mechanisms through which depressed mood may lead to these biases are not fully understood.

We adopted the hypothesis that emotional feelings (e.g., pleasantness/unpleasantness) of cues serve as a source of information when making judgements^19,20^. Importantly, this hypothesis predicted that when no objective information was available for the disambiguation individuals had to rely on their subjective feelings of the cue to make judgement. In this case, a negative mood state led to the mood-congruent judgement of fully ambiguous cues. In contrast, subjective emotional feelings do not necessarily affect the judgement of unambiguous or less ambiguous cues as objective information (feedback or physical features) is available. Indeed, the sad group and the neutral group did not differ in the accuracy of judging the two unambiguous reference cues (Rt and Pt), suggesting that the two groups had a similar ability in processing physical features of auditory cues and in learning associations between cues and reward/punishment. However, when presented with the fully ambiguous cue (Mt), participants exposed to the sad (but not the neutral) induction held a negative judgement bias comparable to that in the dysphoric group, demonstrating a causal effect of depressed mood on judgement bias.

The question now arises of whether depressed mood affected judgement bias by altering subjective emotional feelings. As depressed mood is tightly associated with reduced pleasure in usual activities^21^ and diminished positive affects^52^, it can be inferred that all cues are perceived as less pleasant in depressed mood and therefore an ambiguous cue is more likely to be judged as a negative cue. The EMG data supports this theory. The Rt-elicited ZM activity was reduced in the sad group and the dysphoric group as compared to the neutral group, indicating that negative moods diminished the perceived pleasantness of all cues. Subsequent mediation analysis indicated that mood state had an indirect effect on judgement bias through perceived pleasantness. That is, the induced-sad mood participants and the dysphoric participants perceived the cues as less pleasant, and those with reduced pleasantness also made more negative judgements in the face of ambiguous cues.

There was no difference in ZM facial muscle activity in response to ambiguous cues in either the induced-sad mood or the dysphoric group compared to the control (neutral) group. This is most likely due to the fact that significant ZM activity can be robustly observed only when the induced pleasantness is of a sufficient strength (e.g., induced by an unambiguous rewarding cue)^53^. In this case, ambiguous cues would not have the capacity to produce changes in ZM activity between different mood states.

An interesting finding of the present study is that participants exposed to sad mood induction showed a significant bias only when judging the fully ambiguous cue (Mt); while in contrast, the spontaneously dysphoric subjects showed judgement bias in response to both the fully ambiguous cue (Mt) and the less ambiguous near-rewarding cue (NRt). These findings, while preliminary, suggest that the generalization of a negative bias to less ambiguous cues may be an indicator of the pathological course of depressive disorders. Normally, a sad mood is an adaptive reaction to loss/failure; when this adaptive function is exaggerated, depressive disorders may occur^54,55^. Negative biases emerging from a transiently induced sad mood may initially be an adaptive function, as such a cognitive bias may help avoid potential loss or conserve energy^56^. However, if individuals exposed to prolonged depressed mood, it can be generalized to less ambiguous (or even unambiguous) cues and become maladaptive. Indeed, dysphoric participants gained significantly fewer earnings compared to other groups, mainly because of gaining less rewards from the less ambiguous near-rewarding cue (NRt) while not incurring more punishments. An alternative explanation is that unlike the sad mood induction group, dysphoric participants were already in a depressed mood during the training stage of the JBT. The mood states might have impacts on the association learning so that dysphoric participants were less apt to associate reward with tone cues. However, during the training stage, dysphoric participants did not differ from the other two groups in judgement accuracy (Supplementary Fig. S5), indicating that association learning was not significantly affected in the dysphoric group.

Our results might also provide some implications for existing interventions for depression. Previous research has suggested that ambiguity processing biases contribute to depression^57,58^. The present study shows that reverse causality is also possible, especially when depressed mood leads to diminished perceived pleasantness of objects/events. This is in line with the notion that several cognitive biases in depression are associated with deficits in the reward system^18,22^, but further studies in the clinical population are required. Future studies may investigate whether interventions targeting these biases would benefit from combining cognitive trainings with direct interventions for mood and anhedonia (e.g., “Positive Affect Treatment”^22^).

### Limitations

The present study is limited by the small sample size and not being pre-registered. The results regarding the dysphoric group are preliminary and should be interpreted with caution, although the main focus of the present study has been the two groups that underwent mood manipulations. The final sample size of the dysphoric group in the EMG analysis is larger than previous facial EMG studies e.g.,^35,59^, but independent replications with pre-registration in a larger sample will be required to strengthen the claims that naturally occurring depression differs from transiently induced depressed mood by overgeneralizing negative ambiguity processing bias.

Using a mediation analysis, we have demonstrated that the negative bias resulted from depressed mood is associated with diminished perceived pleasantness (as indicated by the ZM activity). However, it is important to bear in mind that this association is cross-sectional and causal conclusions cannot be drawn. Future studies need to further elucidate the causal link between perceived pleasantness and ambiguity processing bias.

Participants in the current study were all college students. Use of this specific population may limit the generalization of the conclusions. In future research, results from the current study should be replicated in a clinical population. On the other hand, the use of such a homogenous sample provides a more controlled context to examine mood effects on ambiguity processing bias. Similarly, another key limitation of the present study is that the Judgement Bias Test employs ambiguous cues lacking the real-world relevance, which, although ideal for our present purpose, might limit its ecological validity.

## Conclusions

For the first time, we demonstrate that depressed mood can give rise to ambiguity processing bias by altering subjective emotional feelings. Induced sad mood (vs. neutral mood) yielded a negative bias with a magnitude comparable to that in spontaneous depressed mood, but only the latter showed a tendency of generalization of the bias. The facial EMG data indicates that this mood-induced ambiguity processing bias is accompanied by diminished perceived pleasantness of cues. Our results suggest that depressed mood may exacerbate negative ambiguity processing biases by affecting the reward system, and interventions targeting these biases might benefit from combining cognitive training with direct interventions for mood and anhedonia.

## Supplementary information

Supple_Mats

## Data Availability

The dataset is available at https://osf.io/zjnsb/.
