# Supplementary material for: Ambiguity Processing Bias Induced by Depressed Mood Is Associated with Diminished Pleasantness
Source: Sci Rep. 2019 Dec 10;9:18726. doi: 10.1038/s41598-019-55277-6 (PMC6904491; doi:10.1038/s41598-019-55277-6)
Supplement: Supplementary file 1 — Supple_Mats [file 41598_2019_55277_MOESM1_ESM.docx]

***Supplementary Materials* for**

**Ambiguity Processing Bias Induced by Depressed Mood Is Associated with Diminished Pleasantness**

**Xiao-Xiao Lin^1,2^, Ya-Bin Sun^1,2^, Yu-Zheng Wang^1,2^, Lu Fan^1,2^, Xin Wang^1,2,3^, Ning Wang^1,2^, Fei Luo^1, 2^, Jin-Yan Wang^1, 2^***

*1. CAS Key Laboratory of Mental Health, Institute of Psychology, Beijing, China*

*2. Department of Psychology, University of Chinese Academy of Sciences, Beijing, China*

*3. Sino-Danish Center for Education and Research, Beijing, China*

*Corresponding author: J.-Y. Wang,

CAS Key Laboratory of Mental Health, Institute of Psychology, 16 Lincui Road, Chaoyang District, Beijing 100101, China

Email: wangjy@psych.ac.cn

S1. Detailed Methodology

**Sample size and statistical power**

An a priori power analysis was conducted using G*Power 3.1 to determine sample size. Based on a behavioral pilot study with 46 participants, we concluded that to observe a significant group × tone interaction among three groups in the JBT, a minimum total sample size of 30 (10 for each group) was required (1-β > 0.8).

**Additional details of the Judgement Bias Task.**

To conceal the purpose of the experiment, participants were told that they would be undergoing a hearing test and that they would win payment based on task performance.

**Training stage.** First, participants were presented with the Rt for five times and were instructed to respond by pressing the REWARD button. After that, the Pt was presented for five times and the participants were instructed to respond by pressing the PUNISHMENT button. They were instructed to memorize the emotional meanings of these two tones and then practiced in the formal training stage (15 Rt trials and 15 Pt trials, randomly ordered). Once completed, participants were told how much they had earned (up to 15￥if all rewards were won and all punishments were avoided). Failure to achieve 80% accuracy during training resulted in extra training. Payment was only rewarded during the first training round. Participants who failed to reach 80% accuracy after three repeated rounds were excluded from the study. This exclusion criterion was determined on the basis of previous studies e.g. ([Enkel et al., 2010](#_ENREF_2)) as well as our own piloting. (Data from training stage are reported in Supplementary Fig. S5.)

**Methodological considerations.** There are several methodological considerations need to be elucidated about whether this paradigm actually measures ambiguity processing bias. One concern is whether it is more appropriate to conceptualize the JBT as a measure of decision making under uncertainty rather than ambiguity. Uncertainty and ambiguity are similar but different concepts; uncertainty emphasizes the unpredictability of future events, while ambiguity emphasizes the ambiguous feature in the present ([Grenier, Barrette, & Ladouceur, 2005](#_ENREF_3)). These concepts are difficult to dissociate, as an ambiguous cue inevitably sows uncertainty in the outcomes. However, uncertainty is usually operationalized as different degrees of unpredictability; the probability or the latency of conditional stimulus onset following a cue are varied across trials to make participants feel unpredictable. In other words, in a typical uncertainty design, participants are manipulated to worry about whether ([e.g. Kirschner, Hilbert, Hoyer, Lueken, & Beesdo-Baum, 2016](#_ENREF_5)) or when ([e.g. Herry et al., 2007](#_ENREF_4)) a future event will occur. While in the JBT, there was no feedback for ambiguous cues to strengthen this feeling of uncertainty. Also, participants were not informed of their current earnings until the task was over. Therefore, although this task also involves uncertainty of outcomes, we assert that this is but a result of ambiguity. When deciding whether a tone cue signaled reward or punishment, participants feel uncertain about the outcome only because the cue is ambiguous. A higher degree of ambiguity results in a higher level of uncertainty, as was indicated by longer response latency (Supplementary Table S6). Hence, the JBT mainly reflects the processing of the features of the cues (i.e., ambiguity), rather than the processing of the outcomes elicited by the cues (i.e., uncertainty).

Another concern is that participants might take strategies to maximize earnings obtained, which would confound the assessment of ambiguity processing bias. For example, they might feel like to make more negative responses to the fully ambiguous cue to avoid potential monetary loss. To avoid this, participants were assured that there was no fully ambiguous cue (which is not true), and that there were equal numbers of near-rewarding cues and near-punishing cues. Thus, participants would know that taking strategies could not maximize the earnings; taking a conservative strategy is as likely to lose potential reward as to avoid potential loss, while taking a liberal strategy is as likely to gain potential reward as to incur potential loss. For example, taking an extremely conservative strategy (identifying all ambiguous cues as PUNISHMENT) is the same as taking an extremely liberal strategy (identifying all ambiguous cues as REWARD); the former will miss all rewards (+0￥) and avoid all punishment (-0￥), while the latter will gain all rewards (+36￥) but also incur all punishment (-36￥). Thus, both strategies make the final earnings from ambiguous cues 0￥.

**Internal reliability.** We defined the internal reliability as correlations of judgement bias score (bias score of the Mt) among three blocks. The Cronbach’s alpha of the JBT in current study is 0.74.

**Additional details of Benjamini–Hochberg procedure Using R**

To adjust for multiple comparisons for our statistical analyses, we utilized the “p.adjust” function of R following the online documentation ([www.rdocumentation.org/packages/stats/versions/3.6.1/topics/p.adjust](http://www.rdocumentation.org/packages/stats/versions/3.6.1/topics/p.adjust) ). We chose to use the Benjamini–Hochberg procedure to control the false discovery rate as recommended in ([Cramer et al., 2016](#_ENREF_1)). First, we extract all p-values from our confirmatory analyses and put them into a .txt file. Then we run the following codes:


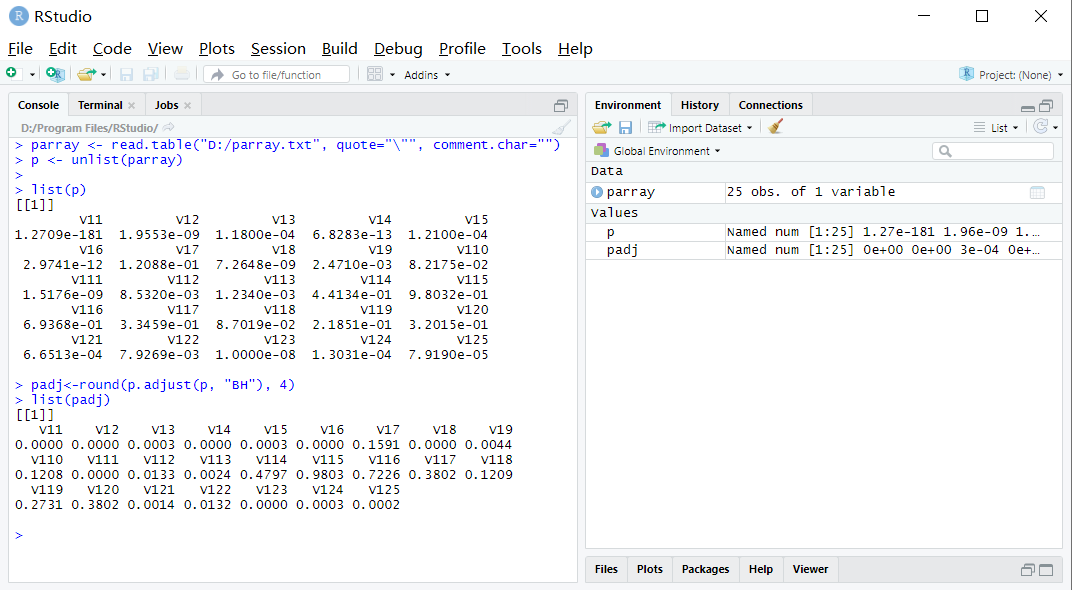


Note: The parameter “BH” stands for the Benjamini–Hochberg method.

S2. Details of post-experiment interviews.

All interviews followed a loosely structured guide. Questions included how they felt about the film, whether they had recovered from that feeling (for sad mood group only), had they ever watched the film clip before, what they thought about the purpose of the experiment, how many new tones they thought had been added in the testing stage, whether they were confident about their judgments for new tones, had they ever participated in psychology study before, their main motivation for participating the experiment (e.g. getting paid, being curious of psychological research, etc.). In addition, dysphoric participants were provided with their BDI-II test results (with interpretations). These participants, especially those reported suicidal ideation, were also advised to seek help from school psychologists.

S3. supplementary ANCOVAs

**Two-way ANCOVA on judgement bias.** To rule out potential confounding effect of demographic characteristics and individual difference in depression-related cognitions, a repeated measures ANCOVA was conducted, with age, gender, RRS, and STAI as covariates. Also, the sad group seemed to experience mood repair during the testing stage (Figure 2), which was different from the dysphoric group. Therefore, mood repair (defined by subtracting mood rating after MI from posttest rating) was also included as a covariate. All main effects and the group × tone interaction remained significant (tone effect, *F*_2.67,331.47_=17.64, *p* < 0.001, $\eta_{p}^{2}=$ 0.13, observed power > 0.99; group × tone interaction, *F*_5.35, 331.469_ = 8.19, *p* < 0.001, $\eta_{p}^{2}=$0.12, observed power > 0.99; group effect, *F*_2,124_=7.34, *p* = 0.001, $\eta_{p}^{2}=$ 0.11, observed power > 0.90).

**Three-way ANCOVA on Facial EMG.** The three-way interaction (*F*_20.61,1071.79_ = 2.07, *p* < 0.01, $\eta_{p}^{2}=$ 0.04, observed power > 0.99) and tone × group interaction (*F*_4.61,239.47_ = 4.61, *p* < 0.001, $\eta_{p}^{2}=$ 0.09, observed power > 0.95) remained significant for the ZM muscle after controlling for gender, age, RRS, trait anxiety, and mood repair in an analysis of covariance (state anxiety was not included, as there were a significant time× state anxiety interaction).

**Two-way ANCOVA for Rt-elicited ZM activity.** There was no substantial change after controlling for age, gender, RRS, STAI (group × time interaction: *F*_8.04,417.92_ = 2.55, *p* = 0.01, $\eta_{p}^{2}=$ 0.05, observed power > 0.90; group main effect: *F*_2,104_ = 6.29, *p* < 0.01, $\eta_{p}^{2}=$0.11, observed power > 0.80; effect of time was not significant).

Supplementary Table S4. Correlations between self-report, behavioral, and facial EMG measures (N=133 if not otherwise specified).

|  | 2 | 3 | 4 | 5 | 6 | 7 | 8 | 9 | 10 | 11 | 12 | 13 |
| --- | --- | --- | --- | --- | --- | --- | --- | --- | --- | --- | --- | --- |
| 1. BDI-II | 0.43** | 0.39** | 0.70** | 0.70** | 0.38** | 0.47** | 0.19* | -0.53** | -0.13 | -0.21* | -0.28** | -0.08 |
| 2. DAS-pre |  | 0.55** | 0.41** | 0.47** | 0.26** | 0.36** | 0.09 | -0.22* | -0.02 | -0.06 | -0.25** | 0.05 |
| 3. DAS-post |  |  | 0.41** | 0.41** | 0.28* | 0.36** | 0.13 | -0.17* | -0.00 | -0.00 | -0.20* | 0.07 |
| 4. State anxiety |  |  |  | 0.79** | 0.33** | 0.45** | 0.12 | -0.53** | -0.09 | -0.14 | -0.15 | -0.17 |
| 5. Trait anxiety |  |  |  |  | 0.24** | 0.38** | 0.04 | -0.49** | -0.11 | -0.21* | -0.15 | -0.15 |
| 6. Rumination |  |  |  |  |  | 0.87** | 0.87** | -0.26** | -0.13 | -0.08 | -0.13 | -0.05 |
| 7. Brooding |  |  |  |  |  |  | 0.52** | -0.32** | -0.15 | -0.08 | -0.13 | -0.07 |
| 8. Reflection |  |  |  |  |  |  |  | -0.13 | -0.08 | -0.05 | -0.09 | -0.01 |
| 9. Mood (baseline) |  |  |  |  |  |  |  |  | 0.32** | 0.30** | 0.25** | 0.29** |
| 10. Mood (after MI) |  |  |  |  |  |  |  |  |  | 0.48** | 0.20* | 0.37** |
| 11. bias Mt |  |  |  |  |  |  |  |  |  |  | 0.45** | 0.56** |
| 12. bias NRt |  |  |  |  |  |  |  |  |  |  |  | 0.20* |
| 13. mean ZM (N=121) |  |  |  |  |  |  |  |  |  |  |  |  |

BDI-II=Beck Depression Inventory-II; DAS=Dysfunctional Attitudes Scale (Short Form); STAI=State-Trait Anxiety Inventory; MI=mood induction; bias Mt=bias score for middle tone; bias NRt=bias score for near-rewarding tone; mean ZM=averaged percentage change of zygomaticus major EMG in 1000 ms after cue onset.

Pearson’s correlation, * *p*<0.05, ** *p*<0.01.

Supplementary Fig. S5

A. Accuracy in the training stage (first round). A two-way repeated measures ANOVA of group (neutral/sad/dysphoric) × tone valence (Reward/Punishment) were conducted for the counts of correct responses, to examine whether the three group differ in differentiating reward tone and the punishment tone. No significant main effect or interaction was found.

B. Rounds of training. A one-way ANOVA of group was conducted to examine whether the three groups differ in how many rounds they practiced to achieve the training criterion. No significance was found.

Supplementary Table S6. Response latency towards different cues in milliseconds (SD in brackets)

|  | cue types | | | | |
| --- | --- | --- | --- | --- | --- |
|  | ***R***t | ***NR***t | ***M***t | ***NP***t | ***P***t |
| Neutral, n=31 | 318.89 (61.79) | 355.80 (56.23) | 369.96 (65.14) | 355.90 (80.74) | 311.66 (66.00) |
| Sad mood, n=34 | 318.03 (61.34) | 343.11 (66.43) | 378.66 (74.62) | 350.86 (69.77) | 322.25 (67.87) |
| Dysphoric, n=16 | 340.63 (66.17) | 376.72 (73.19) | 386.15 (53.57) | 348.58 (78.87) | 326.08 (58.31) |

As in previous studies ([Schick et al., 2015](#_ENREF_6); [Schick, Wessa, Vollmayr, Kuehner, & Kanske, 2013](#_ENREF_7)), different degrees of cue ambiguity were confirmed by response latencies. A two-way ANOVA of group × tone was conducted for response latency revealed a significant tone effect (*F*_3.11,227.16_=15.48, *p*<0.001, $\eta_{p}^{2}=$0.18). Group effect (*F*_2,73_=0.45, *p*=0.64, $\eta_{p}^{2}=$0.01) and group × tone interaction (*F*_6.22,227.16_=0.66, *p*=0.69, $\eta_{p}^{2}=$0.02) were not significant. Bonferroni post-test indicated that response latency increased when a tone got more ambiguous (i.e. Mt>NRt/NPt>Rt/Pt).

Rt: rewarding tone; NRt: near-rewarding tone; Mt: middle tone; NPt: near-punishing tone; Pt: punishing tone.

Reference

Cramer, A. O., van Ravenzwaaij, D., Matzke, D., Steingroever, H., Wetzels, R., Grasman, R. P., . . . Wagenmakers, E.-J. (2016). Hidden multiplicity in exploratory multiway ANOVA: Prevalence and remedies. *Psychonomic Bulletin & Review, 23*(2), 640-647.

Enkel, T., Gholizadeh, D., von Bohlen und Halbach, O., Sanchis-Segura, C., Hurlemann, R., Spanagel, R., . . . Vollmayr, B. (2010). Ambiguous-Cue Interpretation is Biased Under Stress- and Depression-Like States in Rats. *Neuropsychopharmacology, 35*(4), 1008-1015. doi:10.1038/npp.2009.204

Grenier, S., Barrette, A. M., & Ladouceur, R. (2005). Intolerance of uncertainty and intolerance of ambiguity: Similarities and differences. *Personality and Individual Differences, 39*(3), 593-600. doi:10.1016/j.paid.2005.02.014

Herry, C., Bach, D. R., Esposito, F., Di Salle, F., Perrig, W. J., Scheffler, K., . . . Seifritz, E. (2007). Processing of temporal unpredictability in human and animal amygdala. *Journal of Neuroscience, 27*(22), 5958-5966. doi:10.1523/jneurosci.5218-06.2007

Kirschner, H., Hilbert, K., Hoyer, J., Lueken, U., & Beesdo-Baum, K. (2016). Psychophsyiological reactivity during uncertainty and ambiguity processing in high and low worriers. *Journal of Behavior Therapy and Experimental Psychiatry, 50*, 97-105. doi:10.1016/j.jbtep.2015.06.001

Schick, A., Adam, R., Vollmayr, B., Kuehner, C., Kanske, P., & Wessa, M. (2015). Neural correlates of valence generalization in an affective conditioning paradigm. *Behavioural brain research, 292*, 147-156.

Schick, A., Wessa, M., Vollmayr, B., Kuehner, C., & Kanske, P. (2013). Indirect assessment of an interpretation bias in humans: neurophysiological and behavioral correlates. *Frontiers in human neuroscience, 7*.
